# Hole‐Selective Self‐Assembled Monolayers Overlaid on NiO_x_ or PEDOT:PSS for Tin‐Based Perovskite Solar Cells – An Overview of Recent Characterization Methods

**DOI:** 10.1002/chem.202502461

**Published:** 2025-09-27

**Authors:** Donghoon Song, Seung Wook Shin, Hui‐Ping Wu, Eric Wei‐Guang Diau

**Affiliations:** ^1^ Department of Chemical Engineering Sunchon National University 255 Jungang‐ro Suncheon 57922 Republic of Korea; ^2^ Future Agricultural Research Division Rural Research Institute Korea Rural Community Corporation Ansan 15634 Republic of Korea; ^3^ Department of Applied Chemistry and Institute of Molecular Science Center for Emergent Functional Matter Science National Yang Ming Chiao Tung University 1001 Ta‐Hseuh Rd. Hsinchu 300093 Taiwan

**Keywords:** characterization methods, p‐type scaffolds, self‐assembled monolayers, tin perovskite solar cells

## Abstract

Self‐assembled monolayers (SAMs) with high hole‐selectivity have seen growing use in the research realm of perovskite solar cells (PSCs). Even though SAMs can be used solely, their hole‐selective performance is amplifiable on a p‐type underlayer such as nickel oxide (NiO_x_) or poly(3,4‐ethylenedioxythiophene) polystyrenesulfonate (PEDOT:PSS). This is especially prominent in lead‐free tin‐based PSCs. Herein, we review recent relevant publications on hole‐selective SAMs overlaid on NiO_x_ or PEDOT:PSS for tin PSCs. We first examine the characteristic features of the SAMs introduced in each publication. Since SAMs can affect the surface properties, interfacial properties, and perovskite quality, we focus on how these aspects are characterized and which methods are employed. In particular, the employed methods are various spanning computational simulations, microscopy imaging and electrochemical, spectroscopic, and crystallographic measurements. Through this review, we aim to encourage researchers to explore these aspects and methodologies in future research on hole‐selective SAMs for tin PSCs.

## Graphical abstract

Successfully applying self‐assembled monolayers with hole‐selectivity for lead‐free tin perovskite solar cells requires judicious investigation with appropriately powerful characterization methods, in addition to efforts in the development of new materials and processing. We herein review the diverse characterization methods, the results obtained through them, and how to interpret those results.

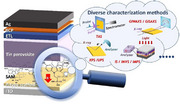

## 1 Introduction

Self‐assembled monolayers (SAMs), formed from numerous molecules to create thin layers in thicknesses ranging from angstroms to a few nanometers, are effective for engineering desired surfaces or interfaces, making them suitable for a wide range of applications and fundamental studies.^[^ ^1^, ^2^, ^3^ ^]^ SAMs are highly appealing due to their up‐scalability, high molecular tunability, and solution‐processability. In particular, SAMs allow conformal deposition onto substrates such as indium tin oxide (ITO) glass even through solution‐processing, whereas other thin layers are typically formed by vacuum‐processing (e.g., atomic layer deposition). Notably, thin layers conformally deposited with high packing density can minimize material waste and thus offer advantages in cost and efficiency compared to those prepared by conventional material deposition techniques.

Recently, SAMs have gained the spotlight in the field of lead halide perovskite solar cells (PSCs) due to their excellent suitability as hole‐selective layers (HSLs), driving efficiencies of power conversion (PCEs) exceeding 26%.^[^ ^4^, ^5^ ^]^ Similarly, SAMs offer a promising outlook for advancing sustainable photovoltaics such as lead‐free halide PSCs.^[^ ^6^, ^7^ ^]^ Among lead‐free perovskites, tin perovskite is a representative candidate for high performance PSCs.^[^ ^8^, ^9^, ^10^ ^]^ The development of tin PSCs incorporating hole‐selective SAMs began in late 2021, achieving a PCE of 6.5% in an inverted planar device architecture.^[^ ^11^ ^]^ This success is attributed to the engineering of SAM interfaces with both the substrate and the perovskite.

In 2024, the efficiency of SAM‐based tin halide PSC (STPSC) was further improved to 9.4%.^[^ ^12^ ^]^ This enhancement is due in large to interfacial engineering at the SAM/perovskite and perovskite/electron selective layer (ESL) interfaces. Notably, SAMs have been reported to significantly influence both the buried and top interfaces of tin perovskite layers.^[^ ^13^, ^14^ ^]^ While the aforementioned PCEs are sub‐10% and achieved with SAM HSLs alone, they have exceeded 14% when SAMs are combined with a p‐type scaffold such as poly(3,4‐ethylenedioxythiophene) polystyrenesulfonate (PEDOT:PSS) or NiO_x_.^[^ ^13^, ^15^ ^]^ Importantly, this progress is owing to the ability of PEDOT:PSS or NiO_x_ to homogenize the surface properties (e.g., the roughness and composition) of ITO glass, as well as their roles in surface passivation and energy level alignment. This improvement is remarkable given the short development history and has helped narrow the gap with the best‐performing TPSCs (∼17%) reported to date.^[^ ^16^ ^]^ Nevertheless, the overall progress in the development of TPSCs remains relatively slow. To further advance these promising PEDOT:PSS/SAM or NiO_x_/SAM HSLs, judicious investigation of SAMs and their interfacial properties, aided by appropriate characterization methods, is pivotal. As part of this effort, this review explores SAM molecules and surface and interface characterization methods that have been applied for STPSCs to date.

## 2 Characterization Methods for Hole‐Selective SAMs in Tin PSCs

A SAM is comprised of numerous molecules (packing density: >20 trillion molecules per cm^2^), which may consist of all identical molecules or two or more different molecules. Each molecule contains three different functional groups: an anchoring group, a spacer group, and a terminal group. Typically, the anchoring group contributes to the formation of a chemical linkage to the substrate, the spacer group affects intermolecular interactions and charge tunneling, and the terminal group determines the key interfacial properties. The explored functional groups in STPSCs are summarized below. For the anchoring group, phosphonic acid, carboxylic acid, siloxane, dicyano group, cyano phosphonic acid, and cyano carboxylic acid have been explored. For the spacer group, hydrocarbon chains, aromatic group, and thiophene group have been adopted. For the terminal group, carbazole group, quinoxaline group, phenothiazine group, triphenylamine group, bithiophene imide group, thienopyrazine group, carboxylic acid, amine group, chlorine group, methyl group, and trifluoromethyl group have been used. Especially, the SAMs with p‐type moieties could have the highest occupied molecular orbital (HOMO) energy level close to the valence band maximum energy level of tin perovskite to modulate hole selection. While our review focuses on the characterization of SAM‐based TPSCs, additional information on the functional groups and their roles and effects can be found in the literature, which may be useful for designing SAM molecules.^[^ ^1^, ^17^, ^18^, ^19^ ^]^

**Figure 1:**
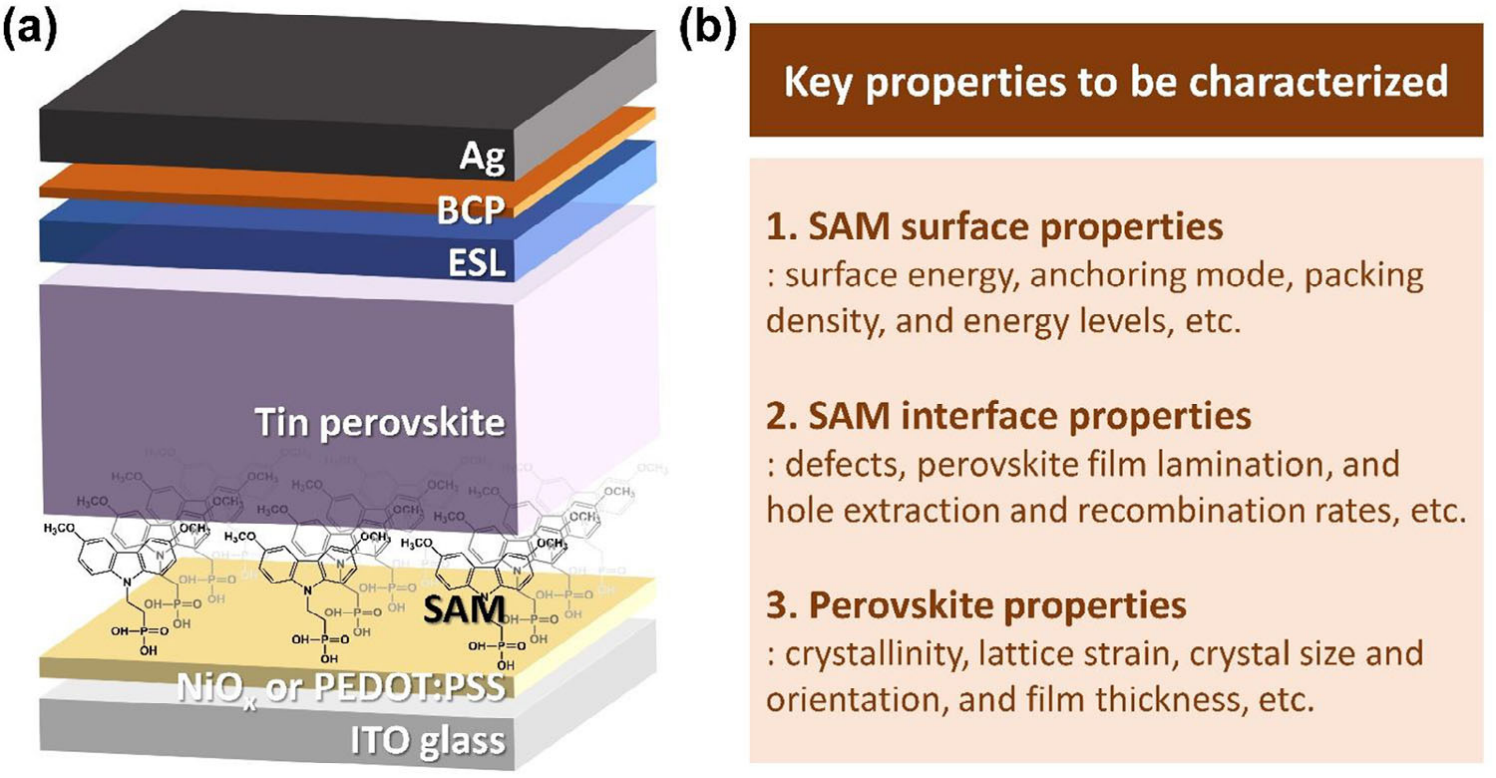
a) Schematic illustration of a hole‐selective SAM‐based tin perovskite solar cell with the structure: ITO/NiO_x_ (or PEDOT:PSS)/SAM/tin perovskite/ESL/bathocuproine (BCP)/Ag. b) Summary of key properties to be characterized in SAM‐based tin PSCs.

Table 1 presents the development history of STPSCs, summarizing the publication date, PCE, and hole‐selective layer. In specific, among eighteen relevant papers,^[^ ^11^, ^12^, ^13^, ^14^, ^15^, ^20^, ^21^, ^22^, ^23^, ^24^, ^25^, ^26^, ^27^, ^28^, ^29^, ^30^, ^31^ ^]^ seven papers have been published with SAMs only while the remaining employed either PEDOT:PSS or NiO_x_ beneath SAMs.^[^ ^13^, ^14^, ^15^, ^20^, ^22^, ^25^, ^27^, ^28^, ^29^, ^31^ ^]^ Overall, high‐performance STPSCs adopt PEDOT:PSS or NiO_x_ as underlays. Both are commercially available and should demonstrate high performance without SAMs for either tin‐based or lead‐based PSCs. Tin perovskites have shallower energy levels than their lead counterparts,^[^ ^32^ ^]^ which could allow competent hole‐selectivity on ITO even without HSLs.^[^ ^33^, ^34^ ^]^ However, ITO could be corroded by halide byproducts from the perovskites layer,^[^ ^35^ ^]^ necessitating the use of HSLs. Even though SAMs can be used alone, PEDOT:PSS or NiO_x_ underlays can complement SAMs to enhance hole‐selective performance,^[^ ^27^, ^36^ ^]^ and vice versa, they can be complemented by SAMs in terms of the hygroscopic and acidic nature (for PEDOT:PSS) and the reactive Ni^≥3+^ species (for NiO_x_) for accelerating perovskite degradation. First, the underlays can reduce substrate roughness which is beneficial for interface contact and perovskite crystallization. It is worth noting that the issue of substrate roughness would play a salient role in STPSCs undergoing rapid crystallization of tin perovskites as well as in the lower interaction strength of SAMs at the buried interface compared with PEDOT:PSS or NiO_x_. Meanwhile, PEDOT:PSS or NiO_x_ could be customized by minimizing parasitic optical and resistive losses without the expense of the surface roughness. Second, they help suppress the formation of SAM defects, which are likely to occur at step edge of ITO or from unbound SAM molecules. Third, especially, the NiO_x_ underlay can augment the density of SAM molecules to improve their surface coverage;^[^ ^36^ ^]^ however, we note that this is affected by combined factors of the substrate surface states, the degree of aggregation of SAM molecules, and the SAM deposition methods. The second and third advantages, which enable the substrate to be effectively hole‐selective, could decelerate recombination, which is crucial for TPSCs suffering from relatively fast recombination. Fourth, the underlays help homogenize hole selection as the ITO surface is heterogeneous due to the presence of various oxide forms of indium and tin. Fifth, the favorable energy‐level alignment is achievable. For example, complete SAM coverage can form staggered gaps at the interfaces of PEDOT:PSS or NiO_x_ with SAMs, thereby enhancing hole selectivity. These advantages can be realized by appropriately tailoring the SAM surface and interfacial properties in addition to perovskite quality (Figure 1), for which diverse related characterization methods have been developed. In this article, we review the papers on STPSCs employing SAMs over an underlay such as PEDOT:PSS or NiO_x_ focusing on the characterization methods used beyond molecular structure analysis. We aim to help readers develop a comprehensive understanding of how each method contributes to evaluating and interpreting interfacial and device performance.

**Table 1:** Summary of the publication history on hole‐selective SAMs, either used alone or in combinatioin with PEDOT:PSS or NiO_x_ for tin PSCs.

Publication date	PCE	SAM only HSL	SAM/PEDOT:PSS HSL	SAM/NiO_x_ HSL	Ref.
2021/12/10	6.5%	Carbazole‐based SAM (MeO‐2PACz)			^[^ ^11^ ^]^
2022/05/15	8.66%		Carbazole‐based SAM (2PACz) on PEDOT:PSS		^[^ ^20^ ^]^
2023/04/25	8.3%	Quinoxaline‐based SAM (TQxD)			^[^ ^21^ ^]^
2023/11/01	12.16%		Carbazole‐based SAM (MeO‐2PACz) on PEDOT:PSS		^[^ ^22^ ^]^
2023/12/01	7.6%	4‐aminobenzoic acid SAM (AB)			^[^ ^23^ ^]^
2023/12/08	5.8%	Carbazole‐based SAM (MeO‐2PACz)			^[^ ^24^ ^]^
2024/01/08	8.6%			Bithiophene imide‐based SAM (BTI‐MN‐b8) on NiO_x_	^[^ ^29^ ^]^
2024/02/12	14.67%		Fluorinated silane‐based SAM (F_3_‐TMOS) on PEDOT:PSS		^[^ ^28^ ^]^
2024/04/12	9.4%	Comolecular SAM (MeO‐2PACz/6PA)			^[^ ^12^ ^]^
2024/04/25	14.19%			π‐extended carbazole‐based SAM (2PADBC) on NiO_x_	^[^ ^15^ ^]^
2024/07/28	10.65%		2‐chloroethylphosphonic acid SAM (CEPA) on PEDOT:PSS		^[^ ^14^ ^]^
2024/09/23	7.7%			Thienopyrazine‐based SAM (TP‐MN) on NiO_x_	^[^ ^27^ ^]^
2025/01/15	8.1%	Triphenylamine‐based SAM (TPAT‐CA)			^[^ ^37^ ^]^
2025/04/07	7.8%	Phenothiazine‐based SAM (PTzBr)			^[^ ^26^ ^]^
2025/04/02	14.7%		π‐extended carbazole‐based SAM (2PADBC) on PEDOT:PSS		^[^ ^13^ ^]^
2025/05/12	8.7%			Triphenylamine‐based SAM (TPA) on NiO_x_	^[^ ^25^ ^]^
2025/07/10	8.65%	Porphyrin‐based SAM (MC‐4)			^[^ ^30^ ^]^
2025/09/01	8.7%			Dithienopyrrole‐based SAM (DTP) on NiO_x_	^[^ ^31^ ^]^

**Figure 2:**
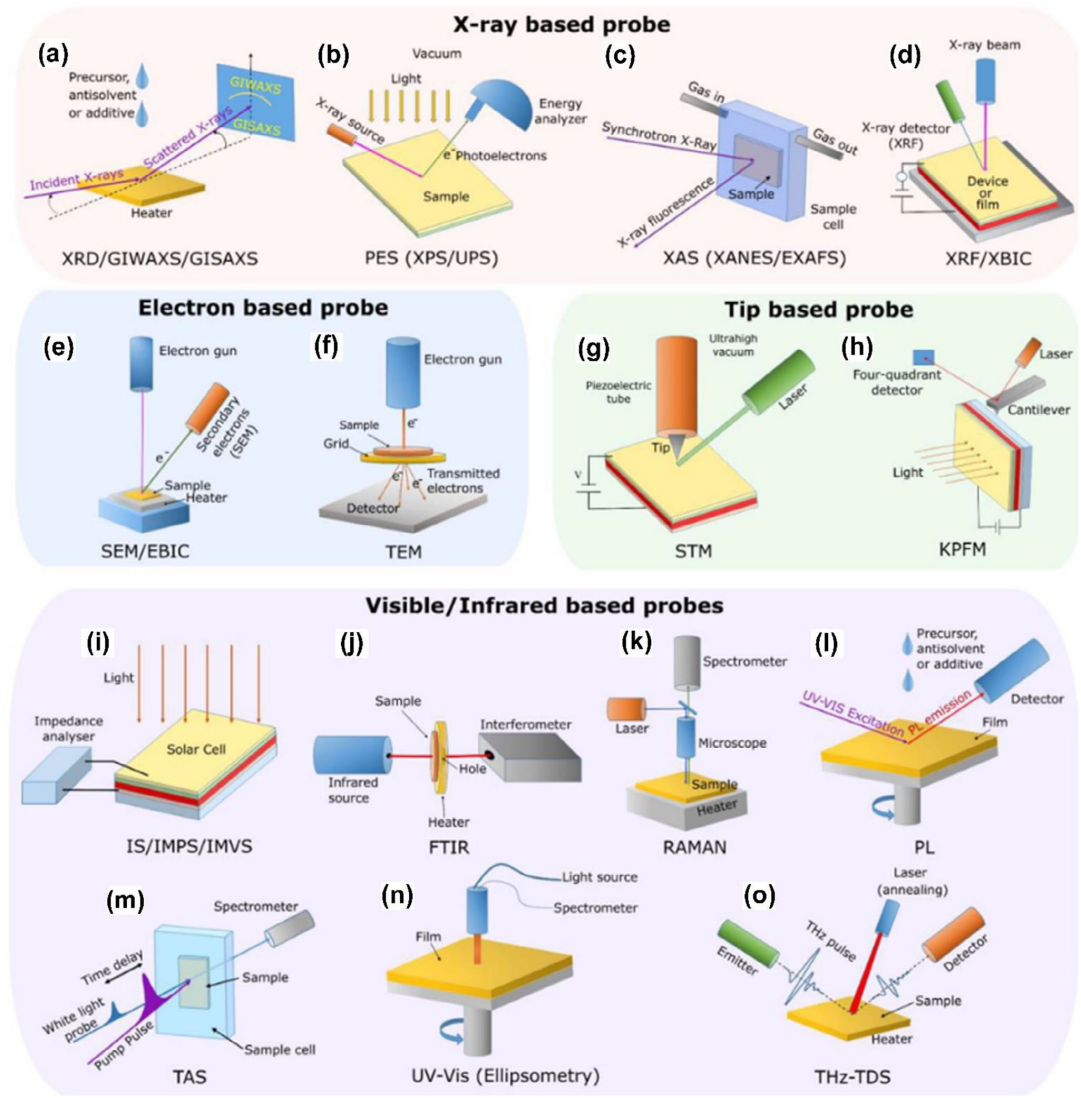
a)–o) Well‐known characterization methods which are applied to PSCs and are also applicable to the investigation of SAM surface and interface properties, as well as perovskite properties in hole‐selective SAM‐based tin PSCs. Reproduced with permission from American Chemical Society, copyright 2023.^[^ ^38^ ^]^

Figure 2 summarizes various characterization methods based on X‐ray, electron‐beam, scanning probe, and light‐based techniques, which are widely used and effective for analyzing components and interfaces in PSCs.^[^ ^38^ ^]^ One can refer to the literature^[^ ^38^ ^]^ for technical details on the application of these methods. In common, these methods are proven surface‐ or interface‐sensitive and appropriate and applicable for investigating the surfaces or interfaces of hole‐selective SAMs (thickness: several nanameters) in TPSCs. Furthermore, electrochemical measurements (e.g., cyclic voltammetry, differential pulse voltammetry (DPV)), nuclear magnetic resonance (NMR) spectroscopy, and molecular simulations have also been utilized (not seen in Figure 2). The following sections discuss the specific types of information that can be obtained from each of these methods.

### 2.1 2PACz SAM on PEDOT:PSS

**Figure 3:**
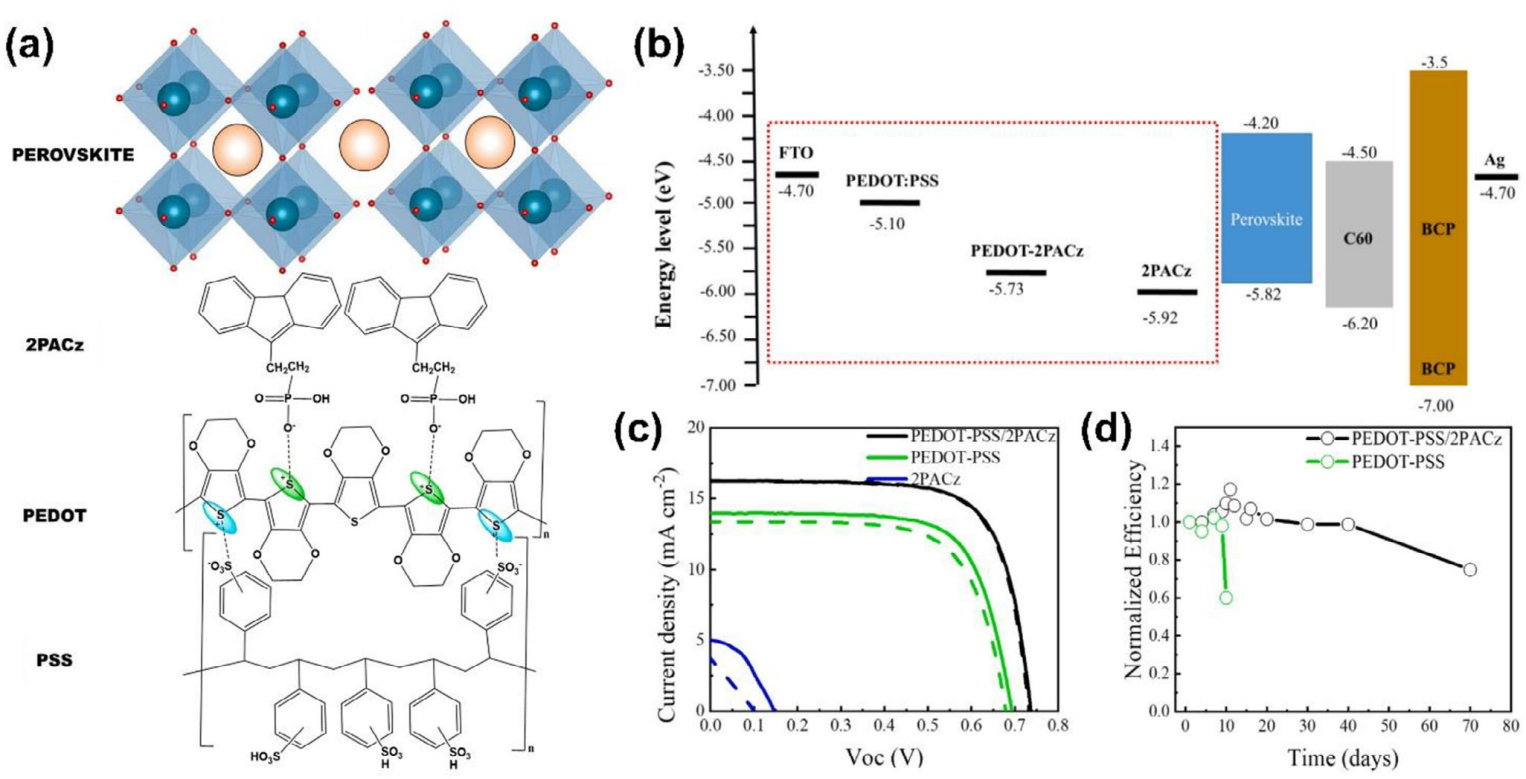
a) Structures of PEDOT:PSS, 2PACz, and perovskite layers. b) Energy diagram (eV, with respect to vacuum) of FTO, various HSLs, perovskite, C60 (buckminsterfullerene), BCP, and Ag. c) Current‐voltage characteristic curves of devices fabricated with various HSLs under illumination of 100 mW/cm^2^. d) Normalized PCE monitored in the dark (without encapsulation). Reproduced with permission from Elsevier, copyright 2022.^[^ ^20^ ^]^

Figure 3a presents the chemical structure of a [2‐(9H‐carbazol‐9‐yl)ethyl]phosphonic acid (2PACz) SAM molecule, which is subjected to anchoring onto PEDOT:PSS using its anchoring group and interfacing with tin perovskite in STPSCs.^[^ ^20^ ^]^ PEDOT:PSS is shown to be an ionic polymer comprised of positively charged PEDOT with S^+^ and negatively charged PSS with SO_3_^−^. SAM molecules can be adsorbed onto PEDOT:PSS by electrostatic force between S^+^ of PEDOT and P‐O^−^ of the deprotonated anchoring group of the 2PACz molecule. Upon the anchoring process, the terminal group of the carbazole moiety in the 2PACz molecule comes into contact with tin perovskite to regulate the interface toward enhanced performance. For example, the valence band maximum energy level of the PEDOT:PSS/2PACz (PEDOT‐2PACz) HSL becomes lowered by more than 0.6 eV (Figure 3b). This lowered energy level and the resulting reduced band offset to the wide bandgap (1.62 eV) perovskite promote hole extraction. As a result, all photovoltaic parameters are reinforced, open‐circuit voltage *V_OC_* (0.68→0.73 V), short‐circuit current *J_SC_* (13.36→16.28 mA cm^−2^), fill factor FF (69→72%), and PCE (6.27→8.62%), as shown in Figure 3c. The minimal forward and backward *J*–*V* hysteresis behavior is accompanied. Meanwhile, it appears that 2PACz significantly contributes to improving device stability likely by passivating the PEDOT:PSS film whose surface is acidic and hygroscopic and capable of degrading perovskites.

### 2.2 MeO‐2PACz SAM on PEDOT:PSS

**Figure 4:**
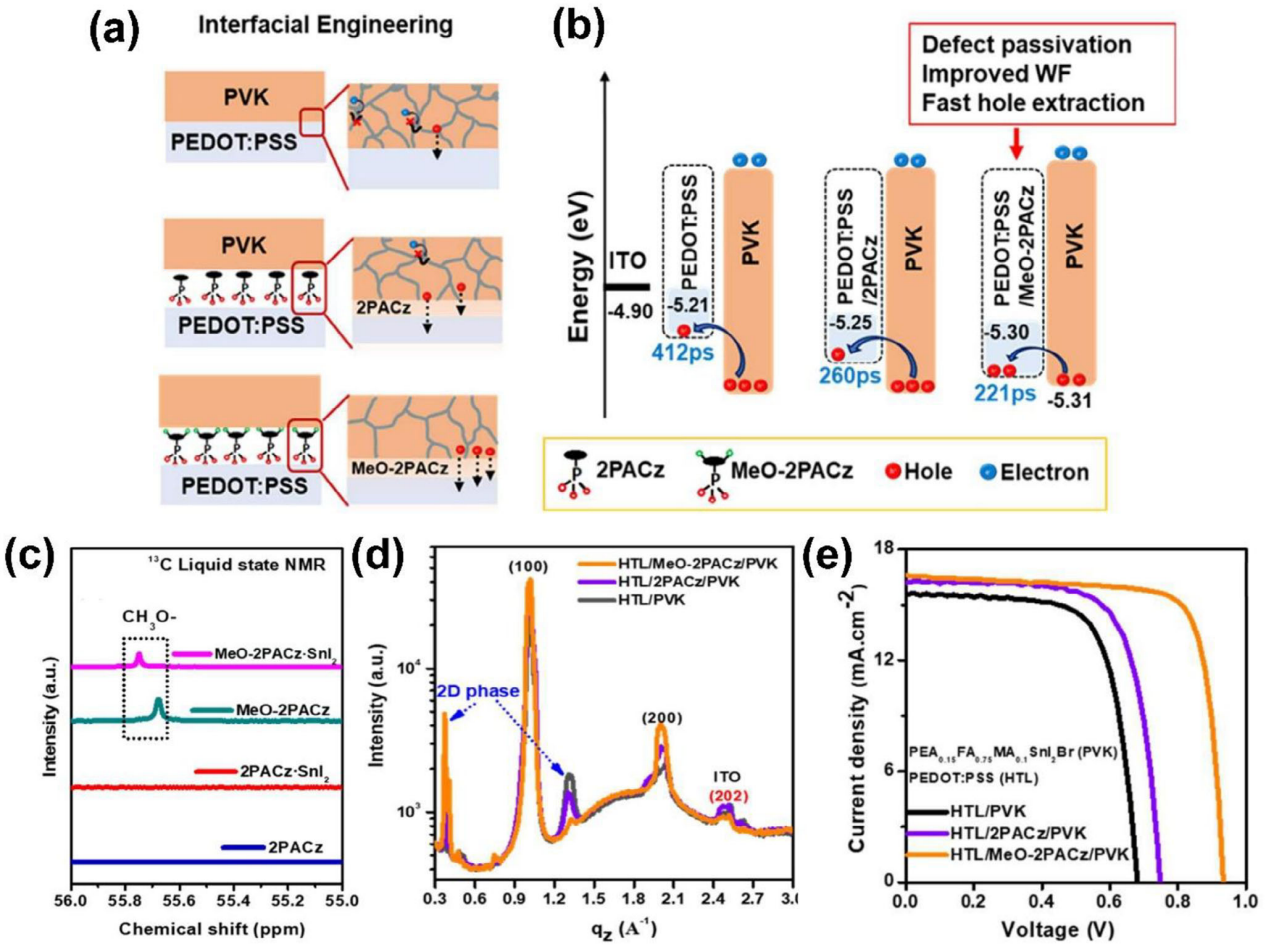
Schematic illustrations of a) interfacial engineering at the PEDOT:PSS, SAM, and perovskite layers and b) changes of work function and energy band alignment of the perovskite with different HSLs. c) ^13^C nuclear magnetic resonance spectra of 2PACz, 2PACz·SnI_2_, MeO‐2PACz, and MeO‐2PACz·SnI_2_ in DMSO‐d_6_ solution. d) Line cut profiles from grazing‐incidence wide‐angle X‐ray scattering images of perovskite with different HSLs. e) Current‐voltage characteristic curves of devices fabricated with PEDOT:PSS/SAM‐based HSLs. Reproduced with permission from Elsevier, copyright 2023.^[^ ^22^ ^]^

[2‐(3,6‐dimethoxy‐9Hcarbazol‐9‐yl)ethyl]phosphonic acid (MeO‐2PACz), which is a member of the 2PACz family and additionally embeds methoxy groups at the 3 and 6 positions, was applied to PEDOT:PSS toward improving hole extraction as Figure 4a shows.^[^ ^22^ ^]^ The hole extraction performance is represented by the hole transport rate in Figure 4b, where PEDOT:PSS/MeO‐2PACz attains the highest hole transport rate (i.e., the smallest time component of 221 ps), which is outputted by femtosecond transient absorption spectroscopy (TAS). We note that the TAS allows exploration of the relaxation kinetics of the photoinduced transient bleach in the perovskite film at different pump fluences and the corresponding lifetime decay profile.^[^ ^22^ ^]^ It is claimed that the effective defect passivation and improved work function account for the superior extraction performance compared to PEDOT:PSS or PEDOT:PSS/2PACz. To probe defect passivation, ^13^C liquid state NMR spectroscopy was carried out with MeO‐2PACz, 2PACz and their mixes with tin(II) iodide. In the NMR spectra displayed in Figure 4c, the signature peak at 55.68 ppm corresponding to the carbon in the methoxy group in MeO‐2PACz is clearly shifted to 55.75 ppm upon its mixing with tin(II) iodide. Figure 4d exhibits the line‐cut profiles of perovskite films deposited onto PEDOT:PSS/MeO‐2PACz and other HSLs. Especially, PEDOT:PSS/MeO‐2PACz demonstrates preferred orientation at the (100) and (200) planes with highly prominent peak intensities. The overall perovskite crystallinity is the best with PEDOT:PSS/MeO‐2PACz according to the X‐ray diffraction measurements. It is therefore claimed that PEDOT:PSS/MeO‐2PACz improves crystal orientation and crystallinity. In Figure 4e, each HSL is compared at a device level. PEDOT:PSS/MeO‐2PACz enhances all photovoltaic parameters relative to others and consequently attains the highest PCE of 12.16%.

### 2.3 BTI‐MN‐b8 SAM on NiO_x_

**Figure 5:**
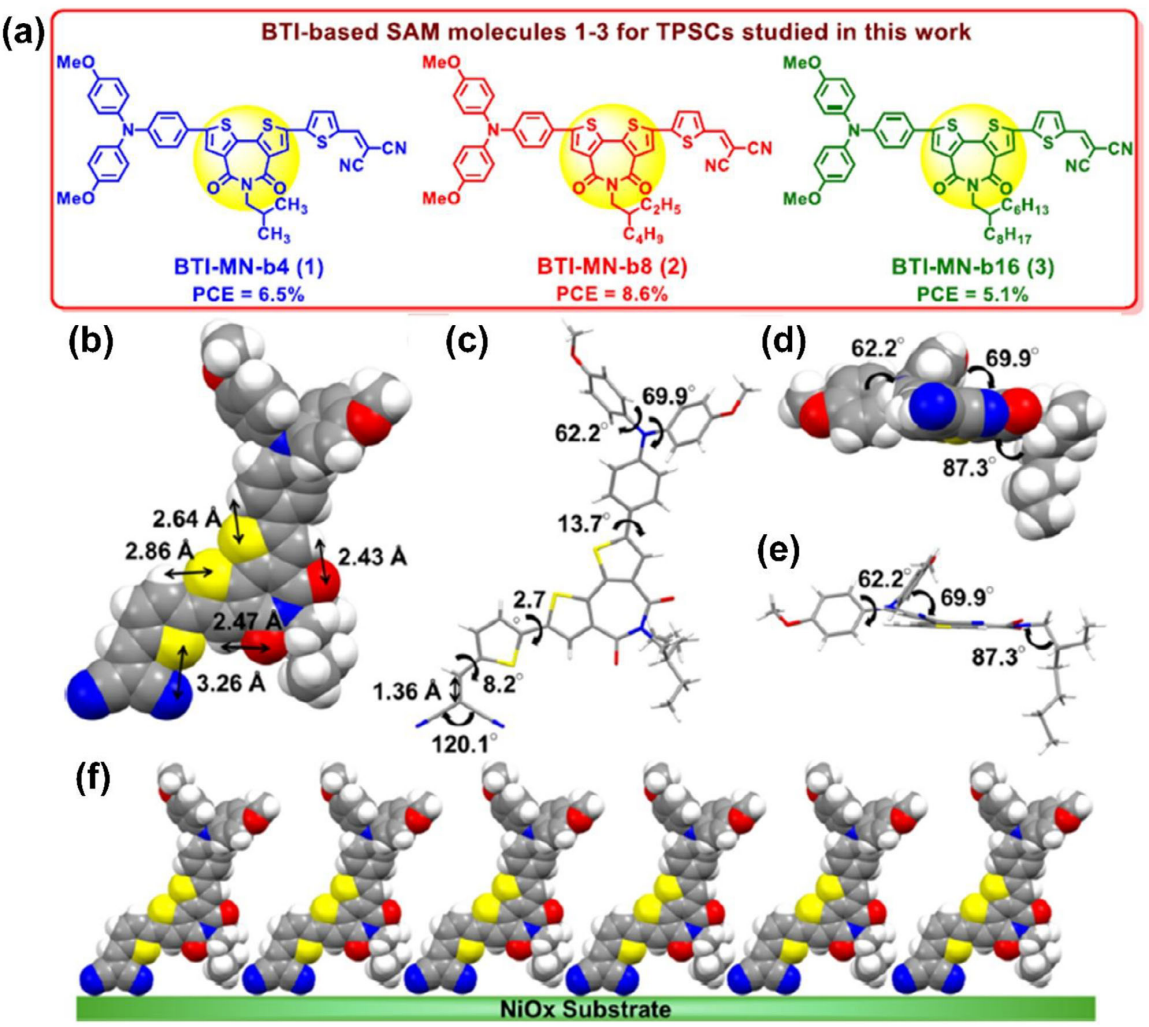
a) Chemical structures of SAM molecules of BTI‐MN‐b4, BTI‐MN‐b8, and BTI‐MN‐b16. (b‐f) Single crystal structure of BTI‐MN‐b8. b) Top view of BTI‐MN‐b8 molecule with intramolecular interactions in a space‐filling model; c) top view of BTI‐MN‐b8 molecule with various twisted angles in stick model; d), e) front views of BTI‐MN‐b8 molecule in the space‐filling and stick models, respectively; and f) expected packing pattern of SAM on NiOx/ITO substrate (front view). Reproduced with permission from American Chemical Society, copyright 2024.^[^ ^29^ ^]^

SAM molecules with the donor moiety of 4,4′‐dimethoxytriphenylamine interfacing the perovskite, which is attached to the bithiophene imide (BTI) moiety, and the BTI moiety is bridged by the thiophene group to the dicyanovinyl (MN) group anchoring to NiO_x_ have been developed.^[^ ^29^ ^]^ This molecule is designated as BTI‐MN‐b8 and its molecular structure is presented in Figure 5a. The alkyl side chains in the bithiophene imide moiety are varied. From atomic force microscopy (AFM), NiO_x_ is found to modify the surface properties (e.g., surface energy and roughness) of the ITO substrate.^[^ ^29^ ^]^ Alkyl chains appear to alter solubility and importantly mitigate aggregation of SAM molecules and control the SAM packing on the NiO_x_/ITO substrate. Especially, the single crystal structure of the best‐performing SAM molecule – BTI‐MN‐b8 (2) – exhibits appropriate dihedral angles and intramolecular interactions and improves π‐conjugation between the bithiophene group and the dicyanovinyl group (Figures 5b–e). It also could facilitate the formation of a SAM with better uniformity and density on the NiO_x_/ITO substrate (Figure 5f), promoting efficient charge transport.

In specific, intramolecular distances of N···S, O···H, and S···H are calculated to be 3.26, 2.43 − 2.47, and 2.64 − 2.86 Å, respectively (Figure 5b), which could promote the formation of highly ordered SAMs on the substrate with a dense and tilted texture. As exhibited in Figure 5c, the good *π‐*conjugation between the BTI and anchoring groups, manifested by the short double‐bond length of C = C (1.36 Å) between the thiophene and MN groups and the relatively small twisted angles of the thiophene spacer relative to the MN (8.2°) and BTI (2.7°), is confirmed, which is desirable for efficient charge transport. Moreover, the phenyl rings in the donor moiety attain the twist angles (62.2° and 69.9°) and improve solubility, easing device fabrication via solution processing. The two cyano groups in the anchoring group are widely angled at 120.1°, which would enable the SAM molecule to stand firmly and perpendicularly on the NiO_x_/ITO substrate. The alkyl chain is attached to the BTI moiety with near perpendicularity (angle: 87.3°). The alkyl chain interacts with the two cyano groups in the bidentate anchoring mode (Figure 5f) whereas it interacts with the BTI in the monodentate anchoring mode. It is noted that moderate alkyl chain lengths can prevent aggregation and packing hindrance of SAM molecules, thereby facilitating the formation of a uniform layer on NiO_x_.

### 2.4 F_3_‐TMOS SAM on PEDOT:PSS

**Figure 6:**
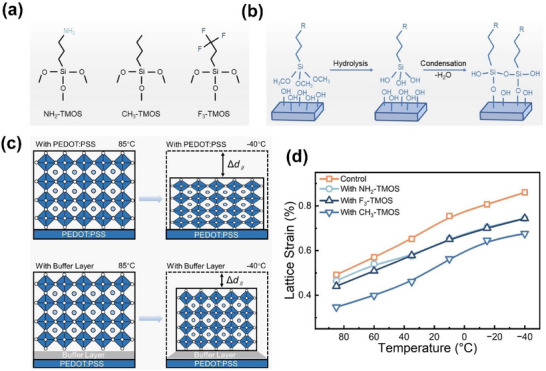
a) The chemical structure of NH_2_‐TMOS, CH_3_‐TMOS and F_3_‐TMOS SAM molecules. b) Schematic diagram of the hydrolysis and condensation reactions of TMOS. c) Schematic diagram of strain formation process and reduction of strain and d) the calculated lattice strain at different temperatures of perovskite films with and without the buffer layers of the SAM molecules. Reproduced with permission from Wiley‐VCH, copyright 2024.^[^ ^28^ ^]^

The SAM molecule whose chemical name is trimethoxy(3,3,3‐trifluoropropyl)‐silane (F_3_‐TMOS) is applied to modify the surface of PEDOT:PSS.^[^ ^28^ ^]^ For comparison, two other SAM molecules coded as NH_2_‐TMOS and CH_3_‐TMOS are explored as well. The chemical structure of all SAM molecules is presented in Figure 6a. In these SAM molecules, the three methoxy groups are commonly embedded, which are hydrolyzed with ease and undergo homo‐condensation (for polymerization) or hetero‐condensation (for anchoring to a hydroxylated surface) as Figure 6b shows. The three SAM molecules are distinct in their terminal groups of ‐NH_2_, ‐CH_3_, and ‐CF_3_, which introduce different molecular dipole moments that affect the energy band alignment and carrier transport. In addition, it is found that the terminal groups can alleviate lattice strain, albeit to different degrees, in the perovskite layer (Figure 6c, d). Specifically, the perovskite deposited on PEDOT:PSS tends to give rise to a large tensile strain mostly along the in‐plane direction (Δ*d_∥_*). This is because the perovskite possesses a positive thermal expansion coefficient and undergoes high temperature annealing and cooling to room temperature leading to its shrinkage. On the other hand, the SAM molecules such as F_3_‐TMOS molecules deposited atop PEDOT:PSS result in the effective regulation of the strain by redistributing it not only along the in‐plane direction but also along the out‐of‐plane direction over a wide range of temperatures (‐40 to 85 °C).

### 2.5 2PADBC SAM on NiO_x_

**Figure 7:**
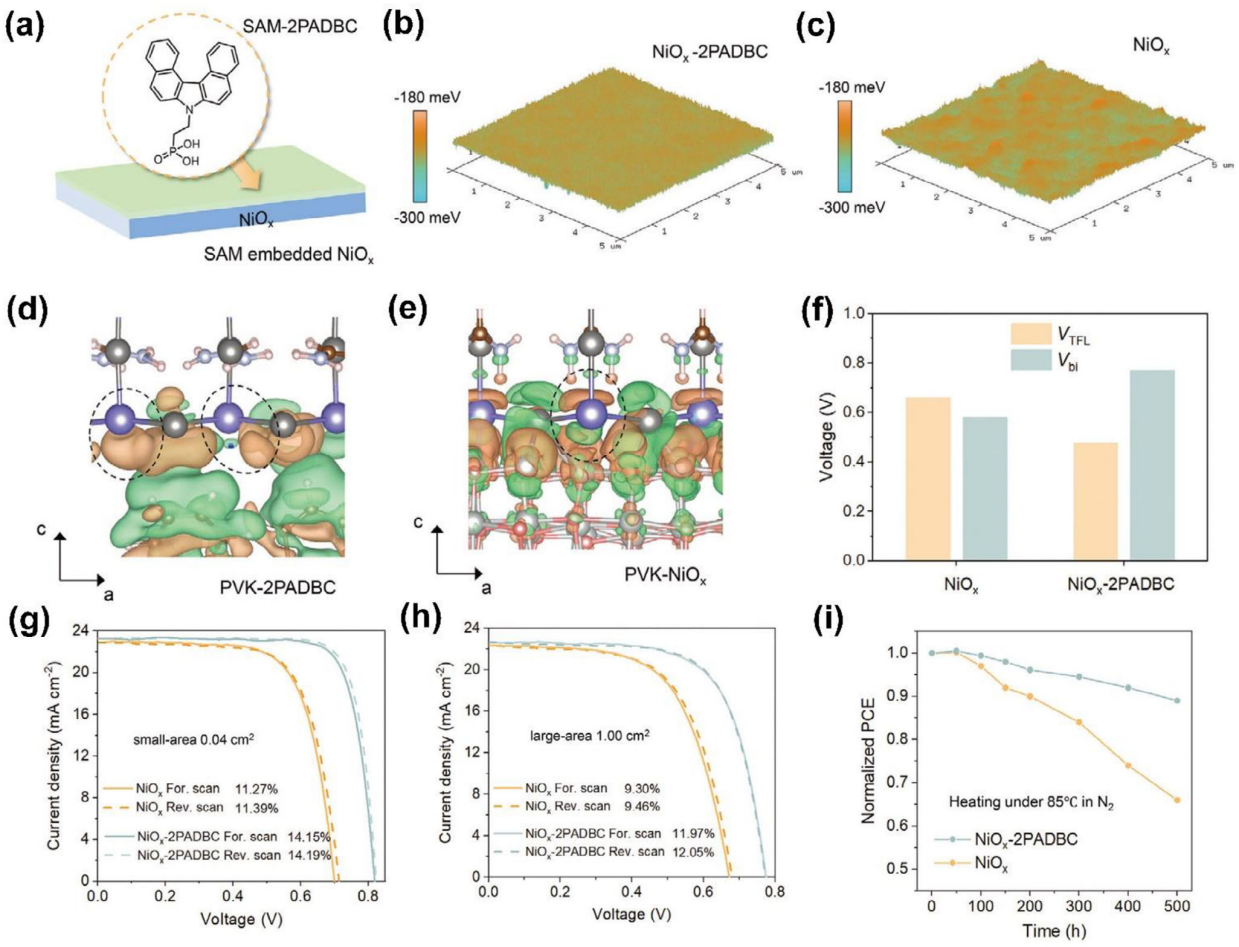
a) Schematic diagram of the SAM‐modified NiO*_x_* film, showing the molecular structure of 2PADBC is presented. 3D KPFM images of the NiO*_x_* films b) with and c) without 2PADBC. Differential charge density simulation of interface of perovskite d) with and e) without 2PADBC. f) Statistics of trap‐filled limit voltage (*V*_TFL_) and built‐in voltage (*V*_bi_) with and without 2PADBC. The *J*–*V* curves of the best‐performing g) small‐area and h) large‐area devices with and without 2PADBC. i) Normalized PCE evolution of unencapsulated devices stored in N_2_ under heating at 85 °C. Reproduced with permission from Wiley‐VCH, copyright 2024.^[^ ^15^ ^]^

(4‐(7H‐dibenzo[c,g]carbazol‐7‐yl)ethyl)phosphonic acid (2PADBC) is a SAM molecule that adds π‐extension to the mother molecule of 2PACz as displayed in Figure 7a.^[^ ^15^ ^]^ The phosphonic acid anchoring group can protect tin perovskite from reactive Ni^≥3+^ defects by sitting on the undercoordinated Ni cations. Applying 2PADBC on NiO_x_ homogenizes the surface potential as evidenced by the 3D Kelvin probe force microscopy (KPFM) images shown in Figure 7b, c. In addition, the 2PADBC molecule serves as an electron donor and enhances the electron density of perovskite, concomitantly increasing the activation energy for Sn oxidation (Figure 7d). This is in stark contrast to the NiO_x_ directly bonded to Sn, which shows the attenuated electron density (Figure 7e).

Two key parameters relevant to the trap state density are trap‐filled limit voltage (*V_TFL_*) and built‐in voltage (*V_bi_*). Figure 7f shows that, with the 2PADBC SAM, *V_TFL_* decreases from 0.72 to 0.52 V and *V_bi_* increases from 0.58 to 0.77 V. This result indicates that the defects such as tetravalent Sn defects are suppressed and additionally the carrier separation is promoted, concomitantly reducing the nonradiative recombination. Figures 4a and 4b show the *J*–*V* curves, evaluated under forward and backward scans, of the best devices with a small area of 0.04 cm^2^ and a large area of 1.00 cm^2^, respectively. In both cases, *V_OC_* and FF are prominently enhanced and the *J*–*V* hysteresis is minimal as compared to the control device. Hence, PCE reaches 14.19% for the small‐area device and 12.05% for the large‐area device. While heating may accelerate the formation of undesirable surface species of Ni^≥3+^ on NiO_x_ in the presence of perovskite, 2PADBC likely inhibits this as shown in Figure 7i where the greater thermal stability of the 2PADBC‐based device is presented with merely a 10% decay at 85 °C for 500 h under a N_2_ atmosphere whereas the control device results in more than 30% decay.

### 2.6 CEPA SAM on PEDOT:PSS

**Figure 8:**
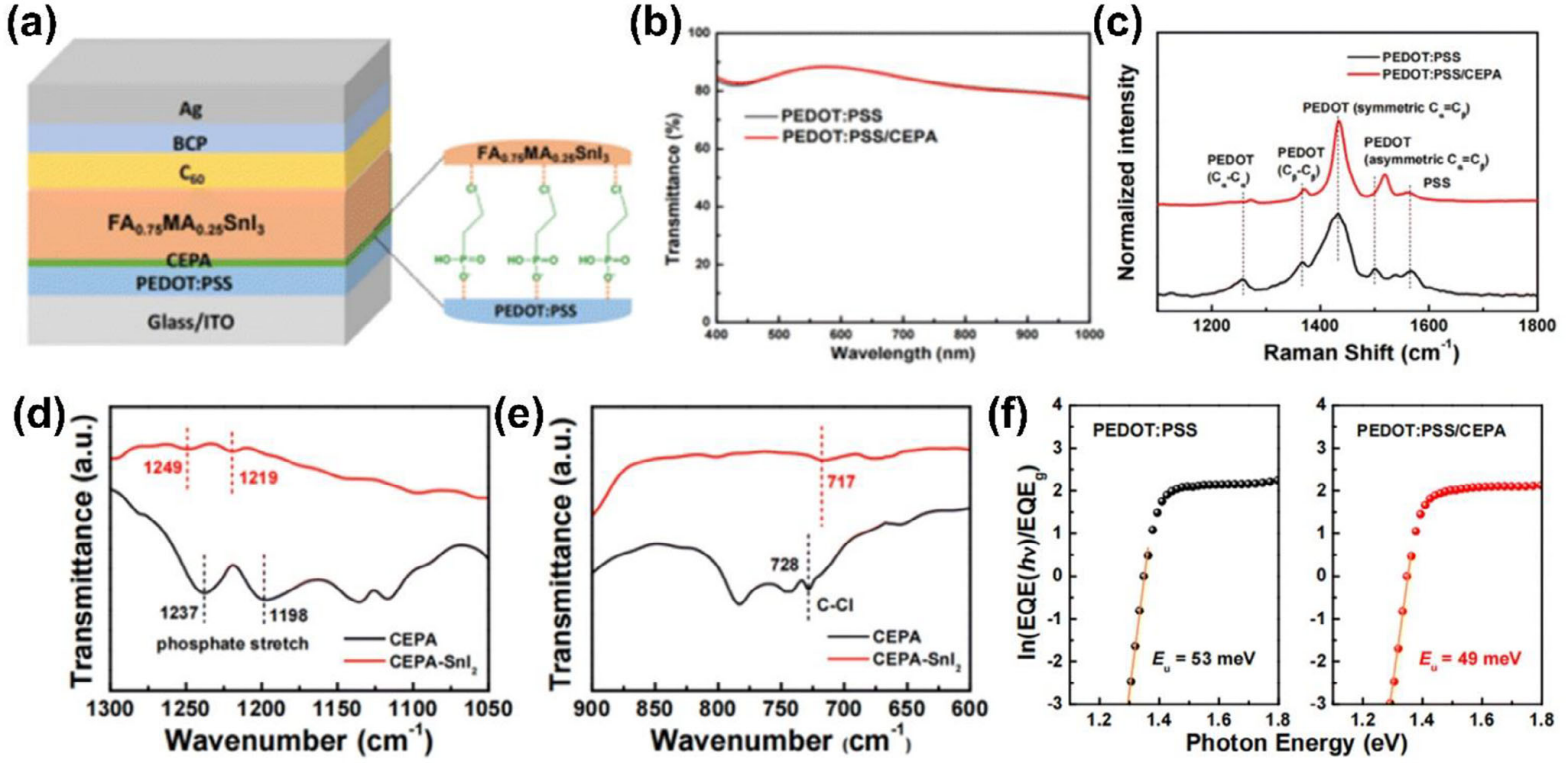
a) Schematic of the device based on CEPA SAM‐modified PEDOT:PSS. b) Transmittance and c) Raman spectra of PEDOT:PSS films with and without CEPA modification. FTIR spectra of d) the phosphate and e) C–Cl functional groups of CEPA and CEPA–SnI_2_ films. f) Urbach energy (*E_U_*) calculation using the EQE spectra of Sn‐PSCs based on PEDOT:PSS and PEDOT:PSS/CEPA films. Reproduced with permission from Royal Society of Chemistry, copyright 2024.^[^ ^14^ ^]^

The SAM molecule of 2‐chloroethylphosphonic acid (CEPA) whose chemical structure is presented in Figure 8a is coated onto PEDOT:PSS.^[^ ^14^ ^]^ The chlorine terminal group modifies PEDOT:PSS to increase hydrophobicity. Importantly, it is interactive with tin perovskite. As revealed by scanning electron microscope (SEM) imaging and photoluminescence (PL) mapping, this suppresses the formation of voids and impurity phases to reduce interfacial defects at the buried interface.^[^ ^14^ ^]^ Desirably, the CEPA modification on PEDOT:PSS does not give rise to a loss in light transmittance over the visible and near infrared light absorption range, Figure 8b. The Raman spectra exhibited in Figure 8c verify that CEPA molecules are chemically anchored onto PEDOT:PSS. Specifically, the peaks at 1258, 1367, 1431, and 1502 cm^−1^ emerge from the symmetric stretching vibration of C_α_‒C_α′_, stretching deformation of C_β_‒C_β_, and symmetric and asymmetric stretching vibrations of C_α_ = C_β_ in PEDOT, respectively. In particular, the symmetric vibration band is found to be narrower and red‐shifted upon CEPA modification. This change implies that PEDOT conformation is altered from benzoid to quinoid, boding well for hole extraction and interfacial defects reduction. In addition, the peaks at 1539 and 1566 cm^−1^ attributable to the phenyl group of PSS become weakened likely due to dedoping and decreased PSS upon CEPA modification.

The strong molecular interaction between Sn^2+^ in SnI_2_ and the phosphate group in CEPA is identified in Figure 8d where the spectra obtained from Fourier transform infrared (FTIR) measurements are presented and show that the IR bands peaked at the wavenumbers of 1198 and 1237 cm^−1^ for the phosphate group are shifted to the higher wavenumbers of 1219 and 1249 cm^−1^. The phosphate group can be deprotonated and chemically attached to the exterior of the perovskite crystal. In Figure 8e, the C‒Cl functional group in CEPA is represented by a stretching vibration peak at 728 cm^−1^, which is shifted to a lower wavenumber of 717 cm^−1^ upon interaction with SnI_2_.

Urbach energy can quantify energetic disorder in the band edges of a semiconductor like perovskite. As shown in Figure 8f, Urbach energies are calculated for perovskite films on PEDOT:PSS and PEDOT:PSS/CEPA by fitting the spectra of external quantum efficiency near the absorption onsets. A lower Urbach energy indicates fewer defects in a perovskite film. The calculated Urbach energy is reduced by CEPA from 53 to 49 meV. This reduction comes from the suppression of sub‐gap defects in the perovskite film on CEPA.

### 2.7 TP‐MN SAM on NiO_x_

**Figure 9:**
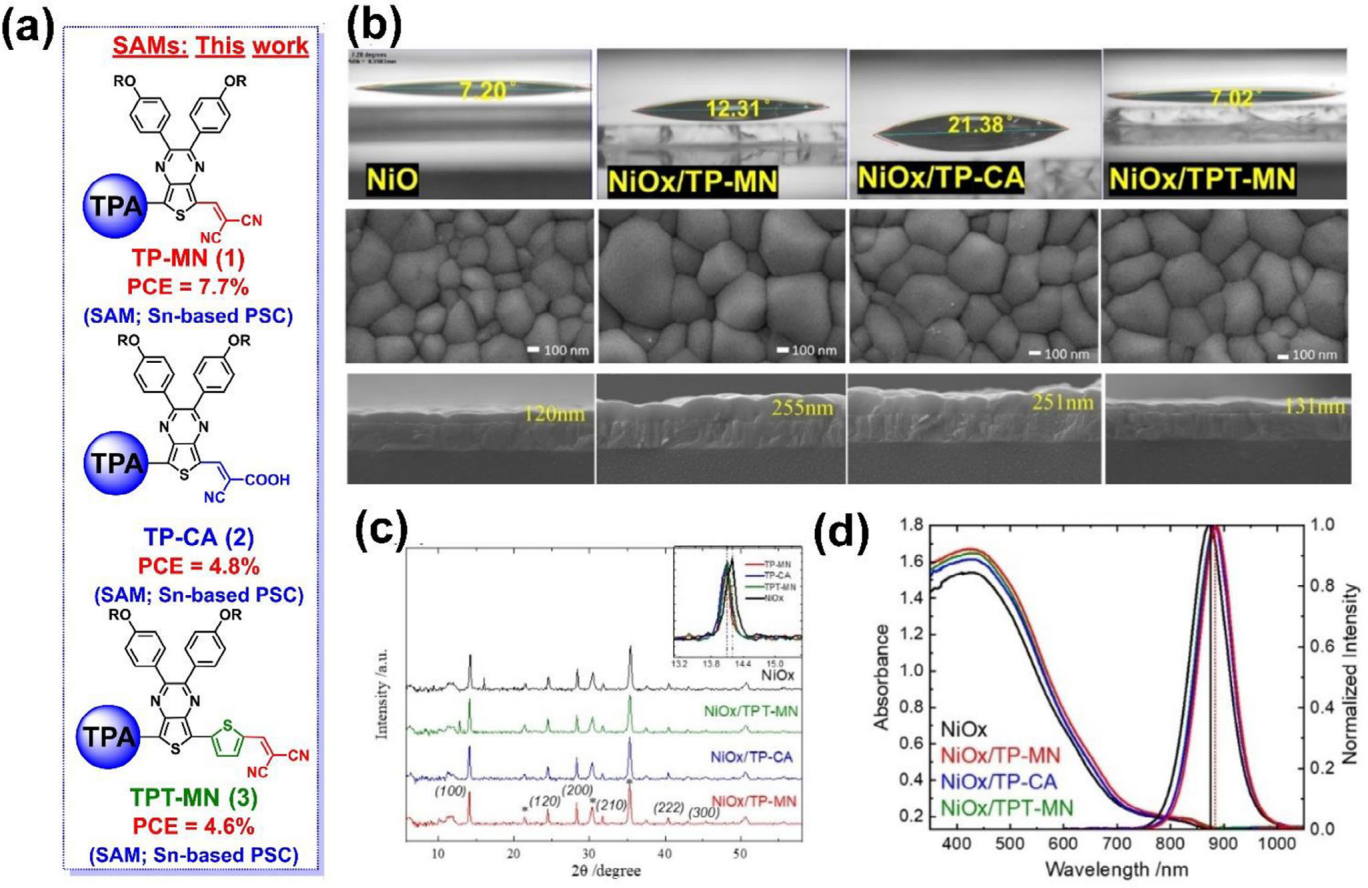
a) Chemical structures of SAM molecules of TP‐MN, TP‐CA, and TPT‐MN. b) Contact angles of the perovskite precursor on the SAM‐modified NiO_x_ substrates in the first row, top‐view SEM images of the resulting perovskite films on the substrates in the second row, and side‐view SEM images of the perovskite films on the substrates in the third row. c) XRD patterns and d) Light absorption and photoluminescence spectra of the perovskite films on the substrates. Reproduced with permission from Wiley‐VCH, copyright 2024.^[^ ^27^ ^]^

The functionalized thienopyrazines (TPs) with varied anchoring moieties are explored as SAM molecules (TP‐MN, TP‐CA, and TPT‐MN) following deposition onto NiO_x_, whose chemical structures are presented in Figure 9a.^[^ ^27^ ^]^ The TP unit is electron‐deficient and is used for the construction of low‐bandgap semiconductors. The TPT unit is a thienopyrazine with π‐extension via a thiophene group. The anchoring group is either CN/CN (MN) or CN/COOH (CA). Interestingly, the anchoring group differentiates the surface energy of the SAM, resulting in a change in the contact angle from 7.20° (for NiO_x_) to 12.31° (for TP‐MN), 21.38° (for TP‐CA), and 7.02° (TPT‐MN) as presented in the first row of Figure 9b. Accordingly, the perovskite film evolves in terms of crystal size and uniformity and cross‐sectional thickness, which are visualized by SEM and shown in the second row and third rows of Figure 9b, respectively. In particular, the perovskite film on TP‐MN shows larger and more uniform crystal grains and increased film thickness (120→255 nm). However, it is arguable whether the contact angle results can be directly correlated with the SEM observations.

According to X‐ray diffraction patterns of perovskite films in Figure 9c, the slight peak shifts take place toward the smaller angles by SAM molecules. It is deduced that the oxidation of Sn^2+^ is in charge of the shifts. This may be corroborated by PL spectra in Figure 9d where the PL peak of pristine NiO_x_ is blue‐shifted. Meanwhile, pristine NiO_x_ results in attenuated absorbance over the entire light absorption range, as shown in Figure 9d, due to the aforementioned lower quality of perovskite.

### 2.8 2PADBC SAM on PEDOT:PSS

**Figure 10:**
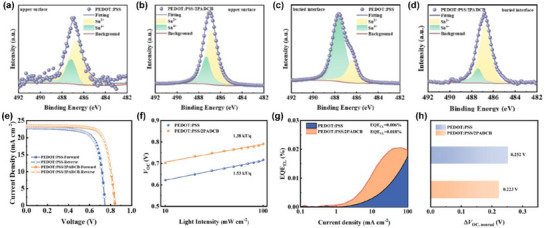
Sn 3d5/2 XPS spectra of a),b) the buried interface and c),d) the upper surface of perovskite films on PEDOT:PSS and PEDOT:PSS/2PADBC SAM films. e) *J *− *V* curves of the devices on PEDOT:PSS and PEDOT:PSS/2PADCB SAM films. f) *V*_OC_ versus light intensity. g) Current density‐dependent electroluminescence‐external‐quantum efficiency spectra and h) the corresponding nonradiative recombination losses (Δ*V*_OC,nonrad_). Reproduced with permission from American Chemical Society, copyright 2025.^[^ ^13^ ^]^

The 2PADBC SAM, which is already used for NiO_x_ as presented in Figure 6, is applied atop PEDOT:PSS, with the attendant merits of protecting tin perovskite and improving the energy levels and the conductivity of PEDOT:PSS.^[^ ^13^ ^]^ In this research, the tin perovskite films are characterized with regards to divalent and tetravalent Sn ions by the Sn 3d5/2 spectra of X‐ray photoelectron spectroscopy (XPS) for their upper surfaces (Figures 10a,b) and buried interfaces, Figures 10c,d, both with and without the 2PADBC SAM atop PEDOT:PSS. The tetravalent Sn ions are defects and the oxidation product of divalent Sn ions. Interestingly, the relative ratio of Sn^2+^ to Sn^4+^ increases by the incorporation of the 2PADBC SAM in both cases. Especially, the ratio increases more prominently at the buried interface (i.e., the HSL/perovskite interface) from 28.57 to 80.85%.

Figure 10e presents the representative *J*–*V* curves, along with the hysteresis behavior, of STPSCs adopting either the 2PADBC SAM or none. The 2PADBC SAM renders a high PCE of 14.7%, which is the best among NiO_x_/SAM‐ or PEDPT:PSS/SAM‐based STPSCs. This is the result of overall increase in photovoltaic parameters, *V_OC_* (0.75→0.84 *V*), *J_SC_* (22.8→23.5 mA cm^−2^), and FF (73.4→74.8%), concurrent with maintaining minimal *J*–*V* hysteresis behavior. The *V_OC_* rise is most outstanding, for which the devices are assessed in terms of the ideality factor (Figure 10f) and electroluminescence external quantum efficiency (EQE_EL_) (Figure 10g). The ideality factors are computed from measurements of devices at various light intensities between 10 and 100 mW cm^−2^ that produce the resulting *V_OC_* values. Their values are 1.38 (for 2PADBC SAM) and 1.58 (for no SAM). The closer the ideality factor value to 1, the better the Shockley‐Read‐Hall recombination (i.e., trap‐assisted recombination) is mitigated. The 2PADBC SAM suppresses this recombination, extending the lifetime of charge carriers and hence enhancing *V_OC_*. Meanwhile, EQE_EL_ measurements under various current densities can offer more detailed picture of *V_OC_*. In specific, the higher EQE_EL_ value (0.018%), with implication of lower nonradiative recombination, with the 2PADBC SAM leads to nonradiative recombination loss of 0.223 V (Figure 10h). In contrast, without the 2PADBC SAM, the EQE_EL_ value is as low as 0.006% to result in the greater recombination loss of 0.252 V.

### 2.9 TPA SAM on NiO_x_

**Figure 11:**
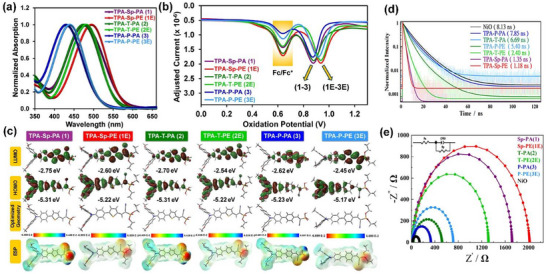
a) UV–vis absorption profiles, b) differential pulse voltammetry plots, c) DFT‐computed energy levels and ESP visualization for SAM molecules of TPA‐Sp‐PA (1), TPA‐Sp‐PE (1E), TPA‐T‐PA (2), TPA‐T‐PE (2E), TPA‐P‐PA (3), and TPA‐P‐PE (3E). d) Photoluminescence decay profiles and e) Nyquist plots for the SAMs. Reproduced with permission from Wiley‐VCH, copyright 2025.^[^ ^25^ ^]^

Six new SAM molecules are developed to anchor onto NiO_x_, each with phosphonic acid (PA) or phosphonic ester (PE) as an anchoring group that is linked via a selenophene (SP), thiophene (T), or phenyl (P) unit to a triphenylamine (TPA) terminal group.^[^ ^25^ ^]^ The SAM molecules are dissolved in *o*‐dichlorobenzene and characterized by the UV‐Vis absorption spectra (Figure 11a). As the linker unit is switched from selenophene to thiophene and phenyl, the absorption peaks exhibit a hypsochromic shift. The absorption peaks appear at wavelengths of 475 nm for TPA‐Sp‐PA, 495 nm for TPA‐Sp‐PE, 473 nm for TPA‐T‐PA, 481 nm for TPA‐T‐PE, 430 nm for TPA‐P‐PA, and 439 nm for TPA‐P‐PE. The heteroatoms in the linkers show different abilities to donate lone pairs into the *π*‐system, thus reducing the optical band gap, which accounts for this peak shift. Meanwhile, DPV was carried out on the SAM molecules, through which the HOMO energy level was determined using the oxidation potential (*E_ox_*) in Figure 11b and the relation, *E_HOMO_* = −(4.44 + *E_ox_*); we note that the DPV measurements proceeded with the solutions containing the SAMs and the standard redox couple (Fc/Fc^+^). The determined *E_HOMO_* values are ‐5.32 eV for TPA‐Sp‐PA, ‐5.38 eV for TPA‐Sp‐PE, ‐5.32 eV for TPA‐T‐PA, ‐5.38 eV for TPA‐T‐PE, ‐5.31 eV for TPA‐P‐PA, and ‐5.36 eV for TPA‐P‐PE; we note that the lowest occupied molecular orbital (LUMO) energy level can be further calculated using the absorption onset in Figure 11a. This result suggests that the type of linker is less influential than the type of anchoring group on the HOMO energy level.

Leveraging density functional theory (DFT) calculations, the electronic structures of the six SAM molecules are determined as shown in Figure 11c. First, it is commonly identified that the HOMOs are mostly localized on the triphenylamine units and the LUMOs are mostly found on the linker groups. Second, the HOMO and LUMO energy levels which are calculated by DFT are in good accordance with those from the DPV results. Third, according to the electrostatic potential (ESP) analysis, the phosphonic acid or phosphonate group carries a higher concentration of negative charges, implying a strong interaction with the NiO_x_ and ITO surfaces and potentially benefiting charge transfer. Meanwhile, using impedance spectroscopy whose results are presented in Figure 11e, the additional interfacial property, namely charge recombination, is revealed; we note that the measurements were performed in the dark at the respective open‐circuit voltages. The charge recombination resistance decreases in the order TPA‐Sp‐PE > TPA‐Sp‐PA > TPA‐T‐PE > TPA‐P‐PE > TPA‐T‐PA > TPA‐P‐PA > no SAM molecule (i.e., NiO_x_ only). It is claimed that this result is consistent with the *V_OC_* trend. The STPSC devices were fabricated using a two‐step sequential method, which solved the problem for the rapid crystallization of the perovskite layer using the conventional one‐step method.

### 2.10 DTP SAM on NiO_x_

**Figure 12:**
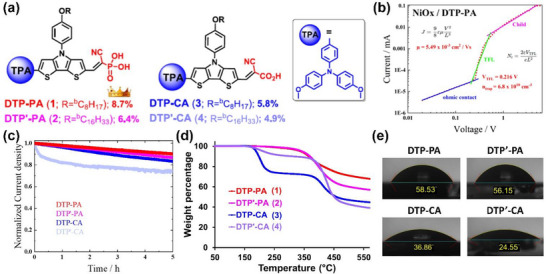
a) Chemical structures and b) TGA curves of SAM molecules of DTP‐PA, DTP’‐PA, DTP‐CA, and DTP’‐CA. c) Water contact angles measured atop the perovskite films. d) Maximum power point tracking diagram of the full devices. e) A current‐voltage plot of the hole‐only device. Reproduced with permission from Elsevier, copyright 2025.

SAMs, which are comprised of a series of donor‐donor‐acceptor type of dithienopyrrole (DTP)‐based molecules (DTP‐PA, DTP’‐PA, DTP‐CA, and DTP’‐CA), have been recently reported, in which the apostrophe represents the elongated aliphatic chain (side functional group; see Figure 12a), PA represents the cyano phosphonic acid (anchoring group), and CA represents the cyano carboxylic acid (anchoring group). While the two different anchoring groups are the major variable, the aliphatic chain is commonly introduced for the solubility of the SAM molecules. On one side, DTP is conjugated with a triphenylamine (TPA) unit in a linear structure. On another side, DTP is linked to an *N*‐substituted phenyl ring for better π‐conjugation extension. While this molecular design is expected to promote efficient hole transfer, the molecular structure is comprehensively verified by NMR spectroscopy and mass spectrometry.

Among them, the DTP‐PA SAM enables the greatest device performance. As evaluated by space‐charge‐limited current (SCLC) analysis, the DTP‐PA SAM exhibits the desired hole‐selective properties, including the highest hole mobility (*μ*) of 5.49 × 10^−3^ cm^2^ V^−1^ s^−1^, the lowest trap‐filling voltage (*V*_TFL_) of 0.216 V, and the lowest trap density (*n*_trap_) of 6.8 × 10^10^ cm^−3^ (see Figure 12b). This contributes to fabricating the STPSC showing a high PCE of 8.7%. The DTP‐PA enables high stability, as demonstrated by a shelf‐storage lifetime of 3600 h (T_∼90_, the time to retain ∼90% of the initial value). This is accompanied by the decent result of maximum power point tracking under constant standard 1 sun illumination (AM 1.5 G, 100 mW cm^−2^, manifested by 5 hours corresponding to the time for the > 90% retention (T_>90_) for the DTP‐PA SAM (Figure 12c). The principal reasons for the stability can be corroborated by thermogravimetric analysis (TGA, Figure 12d) for SAM molecules and water contact angles atop perovskite films (Figure 12e). In the TGA curves, the PA‐containing SAM molecules of DTP‐PA and DTP’‐PA show prominently > 100 °C higher decomposition temperatures (near 340 °C), indicative of higher thermal stability. In the water contact angle images, the PA‐containing SAM molecules of DTP‐PA and DTP’‐PA lead to perovskite films with strengthened hydrophobicity. While more investigation is required to understand these interesting results, the strengthened hydrophobicity could be beneficial for long‐term operation of the devices.

## 3 Summary and Outlook

SAMs constructed on a p‐type scaffold such as PEDOT:PSS or NiO_x_ can be potent for extracting holes in lead‐free tin PSCs. However, their advancement has lagged behind that of other perovskite photovoltaics. To promote further progress, we review characterization methods capable of examining the properties of SAMs and perovskite films, which can guide the development of new types of SAM molecules and the optimization of their interfaces. Specifically, for the surface properties of the SAMs, the surface energy (by the contact angles), HOMO and LUMO energy levels (by DPV or UPS together with light absorption spectroscopy), potential map (by KPFM), roughness (by AFM) and parasitic light absorption (by light absorption spectroscopy) are characterized. In addition, molecular simulation is often explored to predict the optimal geometrical structure, interaction with perovskite, and anchoring onto substrates of SAM molecules. We note that the thickness and packing density (or coverage) of SAMs, essential to evaluate hole‐extraction performance in detail, have been little characterized for tin PSCs. SAM thickness could be evaluated by light absorption spectroscopy,^[^ ^39^ ^]^ ellipsometry^[^ ^36^ ^]^ or XPS.^[^ ^40^ ^]^ In absorption spectra, the absorbance of SAM is presented and it could be used, along with the coverage and the extinction coefficient, for thickness evaluation. Ellipsometry spectra exhibiting the refractive index and the extinction coefficient over the UV‐Vis‐NIR range are output by light reflection, which is often sensitive to substrate roughness. The thickness is verifiable with the substrate element peaks in XPS which are attenuated in the presence of SAMs. While these methods would not be well‐suited to thin SAM films, they can be complemented by AFM being capable of imaging films with a thickness below 1 nanometer.^[^ ^41^ ^]^ SAM packing density could be estimated by transmission electron microscopy^[^ ^36^ ^]^ or cyclic voltammetry^[^ ^42^ ^]^ respectively. In addition, the binding of SAM molecules onto substrates is verifiable with XPS and FTIR.^[^ ^43^ ^]^

The interface properties of the SAMs are characterized by multiple methods. PL spectroscopy or TAS is used to determine the hole transfer rates. Hole recombination is investigated by impedance spectroscopy, light‐illumination‐intensity‐dependent photovoltage, or current‐density‐dependent electroluminescence. The origins of the hole transfer and recombination are revealed in terms of the quality of the buried perovskite (by XPS, XRD, SEM or PL) and/or the interface interaction (by IR, Raman, NMR or molecular simulation). Meanwhile, the quality of bulk and top perovskite – which can eventually affect the hole generation and transfer – can be characterized in terms of crystallinity (by XRD or GIWAXS), crystal size or thickness of perovskite films (by SEM) and/or defects (by XPS or PL). We note that perovskite defects are also quantifiable with Mott‐Schottky analysis^[^ ^44^ ^]^ or thermal admittance spectroscopy,^[^ ^45^ ^]^ both of which are performed at the device level. We further note that, with the resulting devices, impedance spectra, external quantum efficiency spectra, and electrochemical measurement results including *J*‐*V* curves should be conducted to investigate SAM performance at the device level. Even though not all of these methods are necessarily applied, we recommend that researchers make efficient use of them depending on the characteristics of their research. For example, if new types of SAM molecules are synthesized, characterization methods focusing on their material properties on substrates should not be excluded.

## Conflict of Interest

The authors declare no conflict of interest.

## Data Availability

Not applicable – No new data generated.

## References

[1] M. Li , M. Liu , F. Qi , F. R. Lin , A. K.‐Y. Jen , Chem. Rev. 2024, 124, 2138. 38421811 10.1021/acs.chemrev.3c00396

[2] S. A. Paniagua , A. J. Giordano , O. L. Smith , S. Barlow , H. Li , N. R. Armstrong , J. E. Pemberton , J.‐L. Brédas , D. Ginger , S. R. Marder , Chem. Rev. 2016, 116, 7117. 27227316 10.1021/acs.chemrev.6b00061

[3] A. Ulman , Chem. Rev. 1996, 96, 1533. 11848802 10.1021/cr9502357

[4] B. Dong , M. Wei , Y. Li , Y. Yang , W. Ma , Y. Zhang , Y. Ran , M. Cui , Z. Su , Q. Fan , Z. Bi , T. Edvinsson , Z. Ding , H. Ju , S. You , S. M. Zakeeruddin , X. Li , A. Hagfeldt , M. Grätzel , Y. Liu , Nat. Energy 2025, 10, 342.

[5] G. Qu , L. Zhang , Y. Qiao , S. Gong , Y. Ding , Y. Tao , S. Cai , X.‐Y. Chang , Q. Chen , P. Xie , J. Feng , C. Gao , G. Li , H. Xiao , F. Wang , H. Hu , J. Yang , S. Chen , A. K.‐Y. Jen , X. Chen , Z.‐X. Xu , Nat. Commun. 2025, 16, 86. 39747047 10.1038/s41467-024-55523-0 PMC11696008

[6] D. Song , S. W. Shin , H.‐P. Wu , E. W.‐G. Diau , J.‐P. Correa‐Baena , ACS Energy Lett. 2025, 10, 1292.

[7] D. Song , S. Ramakrishnan , Y. Xu , Q. Yu , ACS Energy Lett. 2023, 8, 4162.

[8] M. Pitaro , E. K. Tekelenburg , S. Shao , M. A. Loi , Adv. Mater. 2022, 34, 2105844. 34626031 10.1002/adma.202105844 PMC11469212

[9] J. Cao , F. Yan , Energy Environ. Sci. 2021, 14, 1286.

[10] E. W.‐G. Diau , E. Jokar , M. Rameez , ACS Energy Lett. 2019, 4,1930.

[11] D. Song , S. Narra , M.‐Y. Li , J.‐S. Lin , E. W.‐G. Diau , ACS Energy Lett. 2021, 6, 4179.

[12] D. Song , S. Ramakrishnan , Y. Zhang , Q. Yu , ACS Energy Lett. 2024, 9, 1466.

[13] J. Qu , X. Wang , C. Luo , C. Zeng , H. Zhou , Z. Yang , Z. Zhang , J. Jin , Y. Huang , C. Ding , C. Chen , S. Ren , D. Zhao , ACS Appl. Mater. Interfaces 2025, 17, 19783. 40111404 10.1021/acsami.5c01653

[14] K. Cao , H. Ning , N. Xu , W. Zuo , Y. Zhang , M. Yang , J. Xia , L. Liu , S. Chen , J. Mater. Chem. A 2024, 12, 17444.

[15] B. Li , C. Zhang , D. Gao , X. Sun , S. Zhang , Z. Li , J. Gong , S. Li , Z. Zhu , Adv. Mater. 2024, 36, 2309768. 10.1002/adma.202309768 37971969

[16] D. He , P. Chen , J. A. Steele , Z. Wang , H. Xu , M. Zhang , S. Ding , C. Zhang , T. Lin , F. Kremer , H. Xu , M. Hao , L. Wang , Nat. Nanotechnol. 2025, 20, 779. 40240673 10.1038/s41565-025-01905-4 PMC12181075

[17] T. Wu , M. Zhang , X. Gao , H. Shen , X. Liu , Z. Li , J. Xu , X. Hao , ACS Nano 2025, 19, 24508. 40590263 10.1021/acsnano.5c05601

[18] P. Han , Y. Zhang , Adv. Mater. 2024, 36, 2405630. 10.1002/adma.202405630 38940073

[19] S. Y. Kim , S. J. Cho , S. E. Byeon , X. He , H. J. Yoon , Adv. Energy Mater. 2020, 10, 2002606.

[20] M. Chen , G. Kapil , L. Wang , S. Razey Sahamir , A. K. Baranwal , K. Nishimura , Y. Sanehira , Z. Zhang , M. Akmal Kamarudin , Q. Shen , S. Hayase , Chem. Eng. J. 2022, 436, 135196.

[21] S. N. Afraj , C. Kuan , J. Lin , J. Ni , A. Velusamy , M. Chen , E. W. Diau , Adv. Funct. Mater. 2023, 33, 2213939.

[22] S. Cho , P. Pandey , S. Yoon , J. Ryu , D.‐G. Lee , Q. Shen , S. Hayase , H. Song , H. Choi , H. Ahn , C.‐M. Oh , I.‐W. Hwang , J. S. Cho , D.‐W. Kang , Surf. Interfaces 2023, 42, 103478.

[23] A. Abid , P. Rajamanickam , E. Wei‐Guang Diau , Chem. Eng. J. 2023, 477, 146755.

[24] E. Aktas , I. Poli , C. Ponti , G. Li , A. Olivati , D. Di Girolamo , F. A. Alharthi , M. Li , E. Palomares , A. Petrozza , A. Abate , ACS Energy Lett. 2023, 8, 5170. 38094751 10.1021/acsenergylett.3c02098 PMC10714388

[25] Y. Shih , A. Velusamy , C. Kuan , P. Huang , C. Kuo , D. Zeng , C. Liu , S. Hong , X. Jiang , M. Chen , E. W. Diau , Small 2025, 21, 2500642. 40033895 10.1002/smll.202500642 PMC12067149

[26] A. Abid , A. Velusamy , S. N. Afraj , W. Pervez , T.‐Y. Su , S.‐H. Hong , C.‐L. Liu , M.‐C. Chen , E. W.‐G. Diau , J. Mater. Chem. A 2025, 13, 9252.

[27] C. Kuan , S. N. Afraj , Y. Huang , A. Velusamy , C. Liu , T. Su , X. Jiang , J. Lin , M. Chen , E. W. Diau , Angew. Chem. Int. Ed. 2024, 63, e202407228. 10.1002/anie.202407228 38975669

[28] T. Teng , Z. Su , F. Hu , C. Chen , J. Chen , K. Wang , D. Xue , X. Gao , Z. Wang , Angew. Chem. Int. Ed. 2024, 63, e202318133. 10.1002/anie.202318133 38168100

[29] A. Velusamy , C.‐H. Kuan , T.‐C. Lin , Y.‐S. Shih , C.‐L. Liu , D.‐Y. Zeng , Y.‐G. Li , Y.‐H. Wang , X. Jiang , M.‐C. Chen , E. W.‐G. Diau , ACS Appl. Mater. Interfaces 2025, 17, 952. 39727305 10.1021/acsami.4c15688 PMC11783363

[30] C. Kuan , C. Mai , V. Saravanan , T. Lin , Y. Shih , C. Kuo , M. Tsai , C. Yeh , E. W. Diau , Small 2025, 21, 2504259. 40434250 10.1002/smll.202504259 PMC12243714

[31] R. Balasaravanan , C.‐H. Kuan , Y.‐S. Shih , H.‐L. Cheng , D. Ganesan , S.‐H. Hong , C.‐L. Liu , Y.‐R. Zhong , X. Jiang , M.‐C. Chen , E. W.‐G. Diau , Chem. Eng. J. 2025, 519, 165231.

[32] S. Tao , I. Schmidt , G. Brocks , J. Jiang , I. Tranca , K. Meerholz , S. Olthof , Nat. Commun. 2019, 10, 2560. 31189871 10.1038/s41467-019-10468-7 PMC6561953

[33] D. Song , H.‐Y. Tseng , S. Narra , I.‐H. Tsai , E. Wei‐Guang Diau , Chem. Eng. J. 2023, 464, 142635.

[34] D. Song , L. Y. Hsu , C.‐M. Tseng , E. W.‐G. Diau , Mater. Adv. 2021, 2, 754.

[35] C. Zhan , C. Luo , F. Gao , X. Wang , Y. Li , Q. Zhao , Angew. Chem. Int. Ed. 2024, 63, e202403824. 10.1002/anie.202403824 38727541

[36] N. Phung , M. Verheijen , A. Todinova , K. Datta , M. Verhage , A. Al‐Ashouri , H. Köbler , X. Li , A. Abate , S. Albrecht , M. Creatore , ACS Appl. Mater. Interfaces 2022, 14, 2166. 34936322 10.1021/acsami.1c15860 PMC8763377

[37] S. N. Afraj , C. Kuan , H. Cheng , Y. Wang , C. Liu , Y. Shih , J. Lin , Y. Tsai , M. Chen , E. W. Diau , Small 2025, 21, 2408638. 39548937 10.1002/smll.202408638 PMC11735886

[38] R. Szostak , A. de Souza Gonçalves , J. N. de Freitas , P. E. Marchezi , F. L. de Araújo , H. C. N. Tolentino , M. F. Toney , F. das Chagas Marques , A. F. Nogueira , Chem. Rev. 2023, 123, 3160. 36877871 10.1021/acs.chemrev.2c00382

[39] Y. Lin , Y. Zhang , J. Zhang , M. Marcinskas , T. Malinauskas , A. Magomedov , M. I. Nugraha , D. Kaltsas , D. R. Naphade , G. T. Harrison , A. El‐Labban , S. Barlow , S. De Wolf , E. Wang , I. McCulloch , L. Tsetseris , V. Getautis , S. R. Marder , T. D. Anthopoulos , Adv. Energy Mater. 2022, 12, 2202503.

[40] S. Mariotti , I. N. Rabehi , C. Zhang , X. Huo , J. Zhang , P. Ji , T. Wu , T. Li , S. Yuan , X. Liu , T. Guo , C. Ding , H. Wang , A. Bruno , L. K. Ono , Y. Qi , Energy Environ. Mater. 2025, 8, e12825.

[41] T. He , C. D. Frisbie , ACS Nano 2022, 16, 4823. 35243860 10.1021/acsnano.2c00222

[42] M. Liu , L. Bi , W. Jiang , Z. Zeng , S. Tsang , F. R. Lin , A. K.‐Y. Jen , Adv. Mater. 2023, 35, 2304415. 10.1002/adma.202304415 37487572

[43] S. M. Park , M. Wei , N. Lempesis , W. Yu , T. Hossain , L. Agosta , V. Carnevali , H. R. Atapattu , P. Serles , F. T. Eickemeyer , H. Shin , M. Vafaie , D. Choi , K. Darabi , E. D. Jung , Y. Yang , D. Bin Kim , S. M. Zakeeruddin , B. Chen , A. Amassian , T. Filleter , M. G. Kanatzidis , K. R. Graham , L. Xiao , U. Rothlisberger , M. Grätzel , E. H. Sargent , Nature 2023, 624, 289. 37871614 10.1038/s41586-023-06745-7

[44] D. Song , H. Li , Y. Xu , Q. Yu , ACS Energy Lett. 2023, 8, 3280.

[45] S. Joy , T. Hossain , A. Tichy , S. Johnson , K. R. Graham , J. Phys. Chem. Lett. 2024, 15, 3851. 38557111 10.1021/acs.jpclett.4c00505

